# Prognostic significance of HALP score and combination of peripheral blood multiple indicators in patients with early breast cancer

**DOI:** 10.3389/fonc.2023.1253895

**Published:** 2023-12-12

**Authors:** Zirui Zhao, Lingyun Xu

**Affiliations:** ^1^Graduate School of Dalian Medicine University, Dalian Medicine University, Dalian, Liaoning, China; ^2^Department of Breast Surgery, The Affiliated Changzhou No. 2 People’s Hospital of Nanjing Medical University, Changzhou, China

**Keywords:** HALP scores, breast cancer, prognostic, RFS, ROC curve

## Abstract

**Background:**

To assess the prognostic significance of preoperative hemoglobin, albumin, lymphocyte, and platelet (HALP) score combined with multiple peripheral blood indicators in patients with early breast cancer (EBC).

**Methods:**

A total of 411 patients with early invasive breast cancer underwent breast-conserving surgery or radical surgery at Changzhou No.2 People’s Hospital from January 2015 to December 2020. The cut-off values of HALP, neutrophil-to-lymphocyte ratio (NLR), platelet-to-lymphocyte ratio (PLR), lymphocyte-to-monocyte ratio (LMR), and prognostic nutritional index (PNI) were calculated using the software X-tile. The primary outcomes were recurrence-free survival (RFS), which was analyzed using the Kaplan Meier (K-M) method, while log-rank was used to test the differences between high and low curves. Cox regression analysis was used to analyze the prognostic significance of HALP. Furthermore, the prognostic predictive value of independent prognostic factors was determined using the receiver operating characteristic (ROC) curve.

**Results:**

Low HALP score (P<0.0001), high PLR (P<0.0001), and low LMR (P = 0.0345) were significantly associated with worse RFS. Body mass index (BMI)<24 (P = 0.0036), no diabetes (P = 0.0205), earlier TNM stage (P = 0.0005), and no lymph node metastasis (P = 0.0022) were positively correlated with longer survival HALP scores (hazard ratio [HR] 95% confidence interval [CI]: 0.08 (0.024–0.265), P<0.0001), BMI (HR 95%CI: 0.254 (0.109–0.589), P = 0.001), TNM stage (HR 95%CI: 0.153 (0.041–0.571), P = 0.005), and diabetes (HR 95%CI: 0.259 (0.085–0.785), P = 0.017) were demonstrated as independent prognostic factors by Cox regression analysis. The ROC curves depicted that the two most valuable factors were TNM stage and HALP, and combined independent factors were more accurate in prognostic prediction than any single factor. This further indicated that the TNM stage combined HALP or BMI were more valuable combinations.

**Conclusion:**

The HALP score was an independent prognostic factor for EBC and was significantly associated with worse RFS. This score may predict the probability of postoperative tumor recurrence or metastasis before surgery.

## Introduction

Breast cancer has the highest incidence rate globally, which is increasing annually (1). It has the second highest mortality rate for women worldwide after lung cancer; however, fourth in China after lung, colorectal, and gastric carcinomas. According to statistics (2), the incidence rate and mortality of gastric, liver, and esophageal malignancies among Chinese women have declined; however, other cancer types, including breast cancer, have increased since 2000. Breast cancer, a heterogeneous disease, has rapid metastasis and recurrence; therefore, precision medicine is particularly important for its treatment (3). Treatment and screening methods have emerged, and the precise prognostic factors of breast cancer have also become a debating point in recent years. The clinical evaluation of the prognostic indicators of EBC is mostly based on tumor stage, postoperative pathological type, histological grading, and lymph node metastasis (4). The postoperative survival time can be predicted if we can detect an accurate, economic, convenient indicator.

An immunosuppressive state can occur owing to chronic inflammation and malnutrition, increasing the risk of infection and death in patients with chronic diseases. In recent years, multiple studies have reported the role of peripheral blood biomarkers such as the preoperative neutrophil-to-lymphocyte ratio (NLR) (5–9), platelet-to-lymphocyte ratio (PLR) (10–12), lymphocyte-to-monocyte ratio (LMR) (13–15), and prognostic nutritional index (PNI) (16–20) to explore the relationship between the physiological and disease status and predict disease-free survival (DFS) in patients with cancer to some extent. The prognosis of breast cancer is intricately associated with inflammation. A study (9) on NLR reveals that patients with low pre-treatment NLR exhibited a markedly shorter DFS (HR: 6.97, 95% CI: 1.65-10.55, p = 0.002) and OS (HR: 7.79, 95% CI: 1.25-15.07, p = 0.021) in comparison to individuals with NLR(high). Numerous connections also exist between breast cancer and nutritional factors. A meta-analysis (19) demonstrated that OS and DFS exhibited significant improvements in patients with high PNI and a high controlling nutritional status (CONUT). Furthermore, PNI was identified as an independent prognostic factor for breast cancer.

The hemoglobin, albumin, lymphocyte, and platelet (HALP) score is a new predictive indicator that has emerged in recent years. Chen (21) in 2015 was the first to report that hemoglobin, albumin, and lymphocyte may be positively correlated with cancer prognosis; however, platelet may be negatively correlated. An important indicator of anemia, which is a common disease exacerbated by various inflammatory processes, is hemoglobin level measurement. The nutritional and inflammatory states are typically reflected using albumin levels. Low lymphocyte and high platelet levels may indicate impaired immunity and an increased infection risk. Therefore, combining these four biochemical indicators to predict prognosis seems reasonable and feasible. The value of HALP was calculated using the following formulae: hemoglobin (g/L) × albumin (g/L) × lymphocytes (/L)/platelets (/L). HALP score reflects the tolerance of patients with cancer to tumors quickly and systemically and is characterized by a combination of systemic inflammatory, nutritional, and immune indicators. Many studies have reported a certain predictive value for the survival period of patients with cancer in malignant tumors such as bladder cancer (22), advanced colorectal cancer (23), esophageal squamous cell carcinoma (24), pancreatic cancer (PC) (25), renal cell carcinoma (26), and non-small cell lung cancer (NSCLC) (27). Therefore, this study aimed to explore the prognosis of patients with EBC using preoperative HALP score combined with multiple indicators of peripheral blood like NLR, PLR, LMR, and PNI. It also assessed whether the score can serve as an effective, and economical detection method to provide personalized treatment and early intervention foundation for each patient to better benefit them.

## Methods

### Ethics statement

The study underwent review and received approval from the Ethics Committee of The Affiliated Changzhou No. 2 People’s Hospital of Nanjing Medical University (2018KY039_01). Informed consent was duly acquired from all subjects. All procedures were conducted in adherence to pertinent guidelines and regulations.

### Patient population

**Figure 1:**
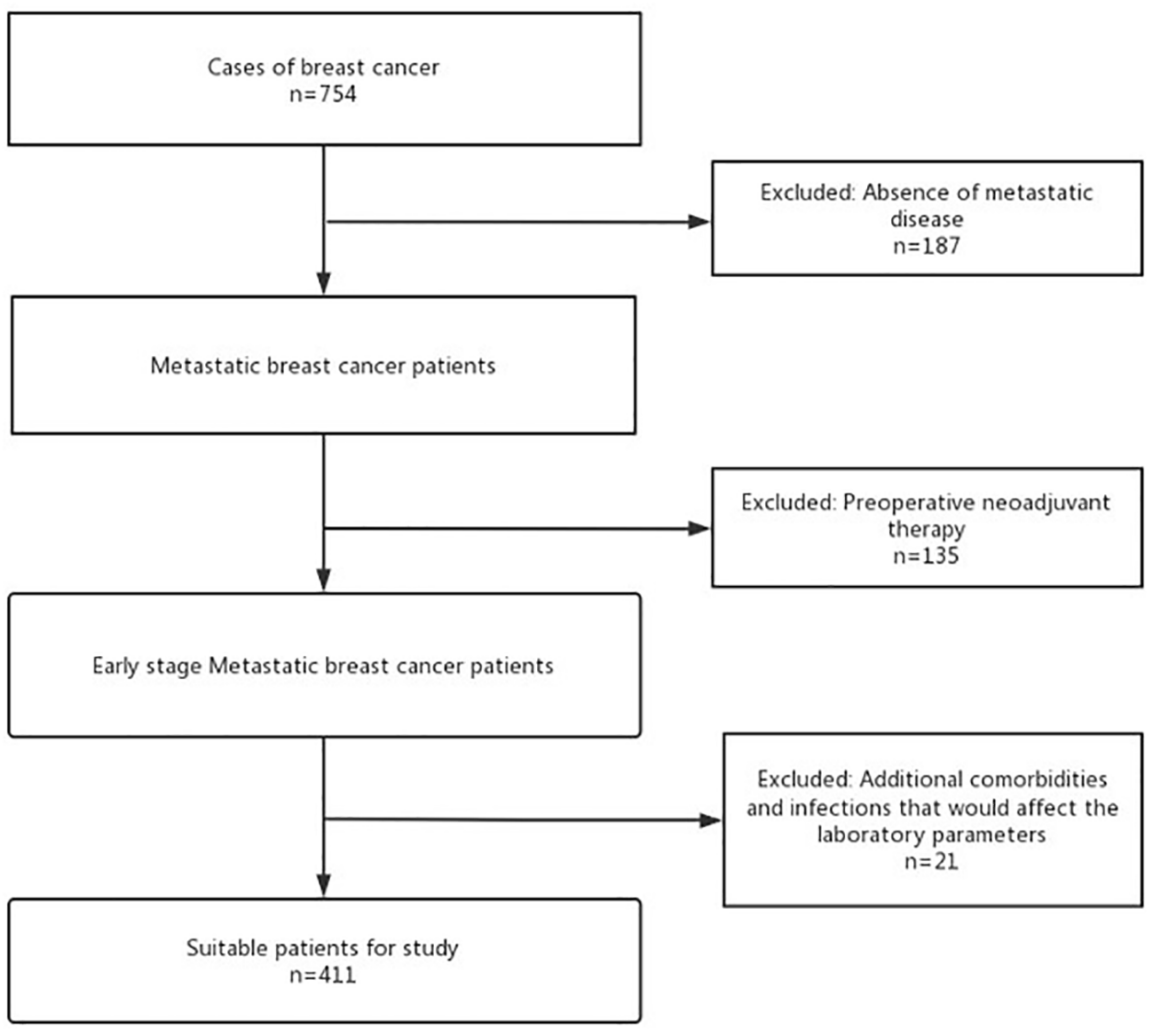
The flowchart of select patient with EBC.

From January 2015 to December 2020, 754 patients with EBC who underwent breast-conserving surgery or radical surgery in Changzhou Second People’s Hospital were screened based on the flowchart (**Figure 1**). Exclusion criteria were as follows: Patients with non-invasive breast cancer or severe heart, liver, kidney, and other major organ failures or significant infections affecting laboratory parameters. Those who received preoperative anti-tumor therapy (including radiation, chemo, targeted, immuno, interventional, and traditional Chinese medicine) were also excluded. Those with incomplete postoperative pathological data recording were also excluded. As depicted in **Figure 1**, a total of 187 non-invasive breast cancer cases, 135 patients who had undergone preoperative therapy, and 21 individuals with significant organ failures were excluded from the analysis. A total of 411 patients with pathologically confirmed early invasive breast cancer were included.

### Data collection

Using the clinical case system by Changzhou Second People’s Hospital to screen enrolled patients and collect clinical data, the following data were included: age, body mass index (BMI), diabetes, menopause, TNM stage, operation, molecular typing, pathological classification, histological grading, lymph node status, immunohistochemistry (including Her-2, ER, PR, Ki-67), adjuvant radiotherapy, and adjuvant chemotherapy Blood biochemical samples were collected from patients one week before surgery and then the HALP, NLR, PLR, LMR, and PNI indices were calculated.

### Definition

The time from the first day after surgery to the recurrence or metastasis of breast cancer was defined as RFS. The HALP, NLR, PLR, LMR, and PNI were calculated using the formulae: HALP =hemoglobin (g/L) × albumin (g/L) × lymphocytes (/L)/platelets (/L); NLR = neutrophil count (n/mm^3^)/lymphocyte count (n/mm^3^); PLR = platelet count (n/mm^3^)/lymphocyte count (n/mm^3^); LMR = lymphocyte count (n/mm^3^)/monocyte count (n/mm^3^); and PNI = albumin level (g/L) + 5 × lymphocyte count (n/mm^3^).

### Follow-up

Outpatient, inpatient, and telephonic follow-ups were used for enrolled patients. Strictly following the National Comprehensive Cancer Network guidelines, follow-up included medical history, physical examination, bilateral breast and axillary lymph nodes B ultrasound, breast molybdenum target, lung CT, abdominal CT, brain MRI, bone scanning, blood biochemistry, blood routine investigations, and peripheral blood tumor indicators. A comprehensive physical examination was performed every 3–6 months in the first 2 years after the operation, then every 6–12 months in the next 3 years until 5 years after the operation, and then once every 1 year till recurrence or metastasis. Moreover, the patient was informed to seek medical attention promptly if they felt unwell.

### Statistical analysis

The cut-off values for each ratio were calculated using the software X-tile and were divided into the following two groups based on each cut-off value: high and low. The K-M method and log rank were used to analyze RFS and test the differences between high and low curves, respectively. The prognostic significance of HALP for EBC was analyzed using Cox regression analysis. Furthermore, the prognostic predictive value of independent prognostic factors in patients with EBC was determined using the ROC curve. All research data were statistically analyzed using SPSS 26.0 software and GraphPad Prism version 9.0. Statistical significance was set at P < 0.05.

## Results

### Baseline characteristics

The clinicopathological characteristics of 411 patients with EBC are listed in **Table 1**. The study population’s median age was 54.52 (range:28–98) years, median BMI was 23.6, and median follow-up period was 54 (range:0.4–93) months till December 2022. Among them, 28 patients experienced recurrence or metastasis, and three died, 35.8% (147/411) patients were aged less than 50 years, 7.3% (30/411) had diabetes before the operation, 89.1% (366/411) received adjuvant chemotherapy; 93.4% (384/411) were confirmed Infiltrating ductal carcinoma, and 57.7% (273/411) were found with no lymph node metastasis. Luminal B (243/411) was the most common sub-type of molecular breast cancer. Only 15.1% (62/411) of patients were at a higher stage, while those who received breast-conserving or radical resection were almost the same (211 vs. 200). According to the calculated formula, the mean ± SD values of HALP, NLR, PLR, LMR, and PNI were 43.02 ± 16.72, 3.02 ± 3.32, 139.39 ± 64.72, 6.68 ± 14.88, and 50.40 ± 4.78, and the cut-off values were 23.6, 1.6, 195.7, 4.9, and 50.5 calculated using the software X-tile 3.6.1.

**Table 1:** The characteristics of patients with EBC.

Characteristics	N=411
Age(<50/≥50)	147 (35.8%) /264 (64.2%)
BMI( < 24/≥24)	238 (57.9%) /173 (42.1%)
Menopause status(Positive/Negative)	221 (53.8%) /190 (46.2)
Diabetes(yes/no)	30 (7.3%) /381 (92.7%)
Adjuvant chemotherapy(yes/no)	366 (89.1%) /45 (10.9%)
Adjuvant radiotherapy(yes/no)	146 (35.5%) /265 (64.5%)
TNM stage( I / II /III)	156 (38.0%) /193 (47.0%) /62 (15.1%)
Molecular typing(Luminal A/ Luminal B/ Her-2/ TNBC)	66 (16.1%) /243 (59.1%) /47 (11.4%) /55 (13.4%)
Pathological type(Infiltrating ductal/ Other type)	384 (93.4%) /27 (6.6%)
Histology grading(1/2/3 grade)	30 (7.3%) /237 (57.7%) /144 (35.0%)
Lymph node status(N0/N1/N2+)	237 (57.7%) /158 (38.4%) /16 (3.9%)
Operation(Breast conserving/ Radical resection)	211 (51.3%) /200 (48.7%)
ER(-/+)	102 (24.8%) /309 (75.2%)
PR(-/+)	144 (35.0%) /257 (65.0%)
Her-2 (-/+)	273 (66.4%) /138 (33.6%)
Ki-67( < 14/≥14)	87 (21.1%) /324 (78.8%)
Recurrence or metastasis(yes/no)	28 (6.8%) /383 (93.1%)
HALP	43.02±16.72
NLR	3.02±3.32
PLR	139.39±64.72
LMR	6.68±14.88
PNI	50.40±4.78

### Relationship between clinicopathological characteristics and RFS

**Figure 2:**
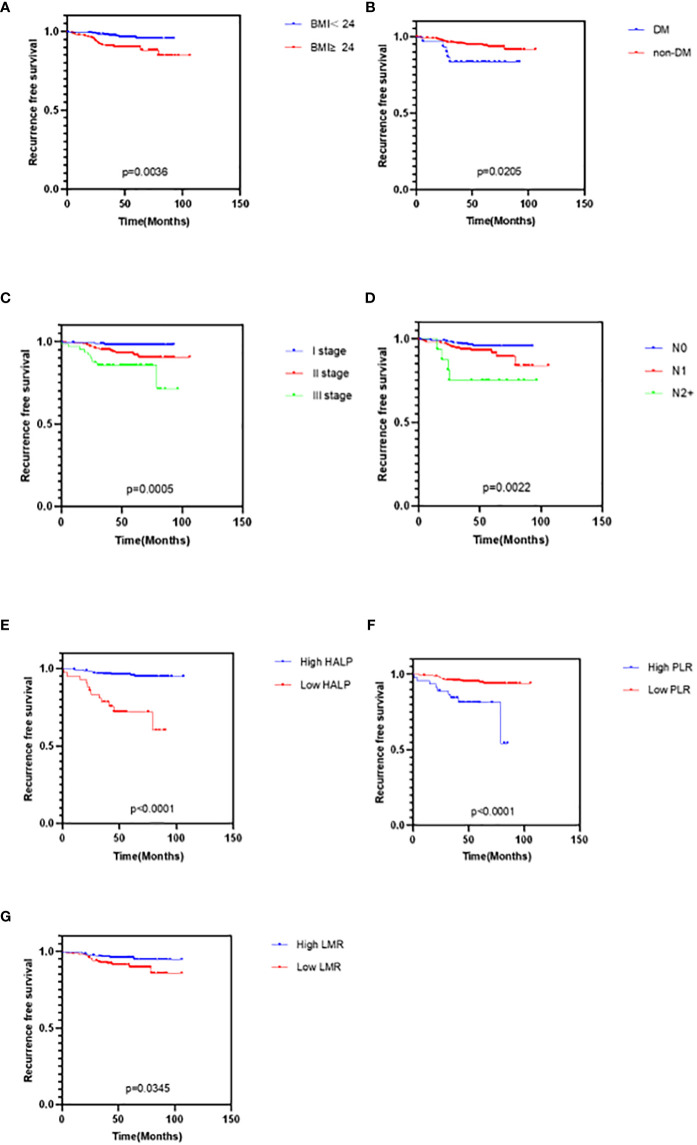
Kaplan-Meier curves for cancer survival in patients with EBC. **(A) **BMI, Body Mass Index **(B)** DM, Diabetes **(C)** TNM stage **(D)** Lymph node status **(E)** HALP, hemoglobin and albumin and lymphocyte and platelet score **(F)** PLR, platelet-to-lymphocyte ratio **(G)** LMR, lymphocyte-to-monocyte ratio.

**Figure 3:**
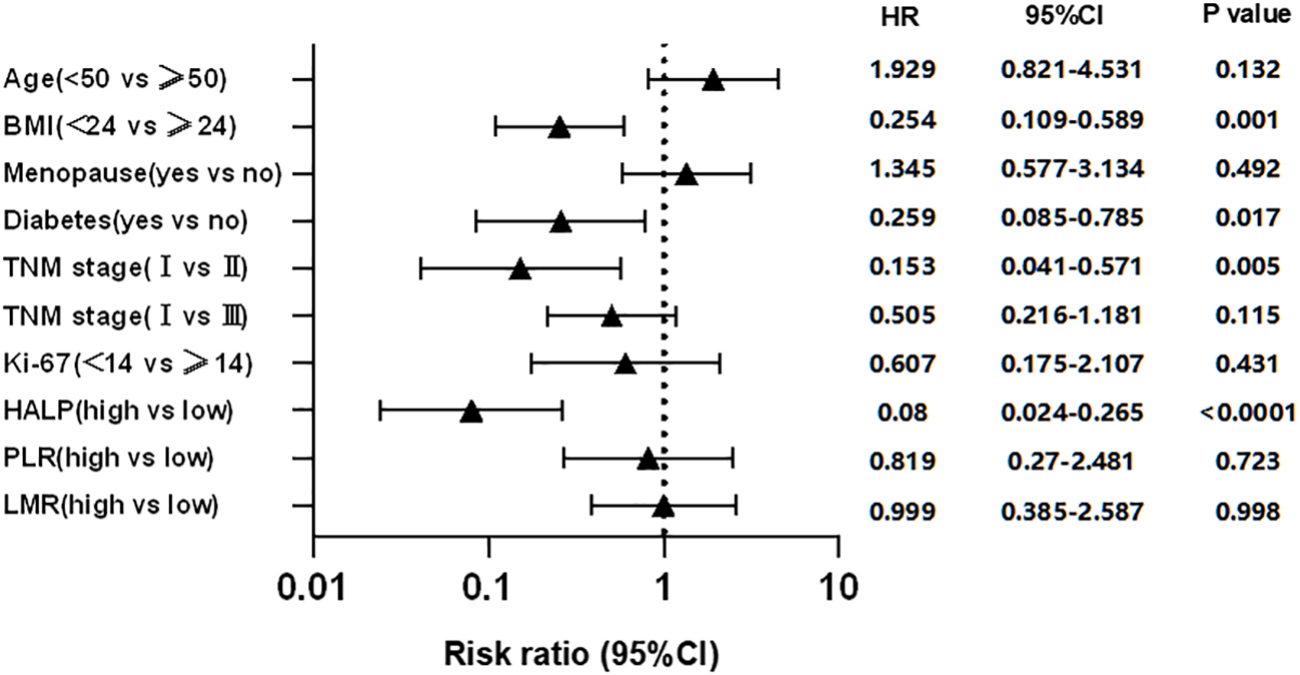
Cox multivariate analysis of factors associated with survival time for EBC patients.

The clinicopathological characteristics were used by Kaplan-Meier curves to analyze survival time. The results demonstrated that diabetes (P = 0.0205), high BMI (P = 0.0036), high TNM stage (P = 0.0005), and worse lymph node status (P = 0.0022) were significantly associated with shorter RFS, while other characteristics were not (**Figure 2**). Cox multivariate analysis demonstrated that diabetes (hazard ratio [HR] 95% confidence interval [CI]: 0.259 [0.085–0.785], P = 0.017), BMI>24 (HR 95%CI: 0.254 [0.109–0.589], P = 0.001), TNM stage (HR 95%CI: 0.153 [0.041–0.571], P = 0.005) were independent prognostic factor for RFS in EBC (**Figure 3**).

### Relationship between HALP, PNI, NLR, PLR, LMR, and RFS

The clinicopathological characteristics between high and low groups of HALP, NLR, PLR, LMR, and PNI are demonstrated in **Supplementary 1**. Approximately 9.5% (42/411) of patients had low HALP (HALP<23.6); 32.2% (142/411) of patients had low NLR (NLR<1.6); 83.0% (366/411) of patients had low PLR (PLR<195.7); 43.3% (191/411) of patients had low LMR (LMR<4.9); and 50.8% (224/411) of patients had low PNI (PNI<50.5). The result depicted no difference between any high and low groups in these characteristics (all P>0.05). Furthermore, we found that patients with low HALP (P<0.0001), high PLR (P<0.0001), or low LMR (P = 0.0345) had shorter survival time; however, NLR (P>0.05) and PNI (P>0.05) were not related to RFS (**Figure 2**). Cox multivariate analysis demonstrated that only HALP (HR 95%CI: 0.08 (0.024–0.265), P<0.0001) was an independent prognostic factor for RFS in EBC (**Figure 3**).

### Prognostic significance of single and multiple indicators

**Figure 4:**
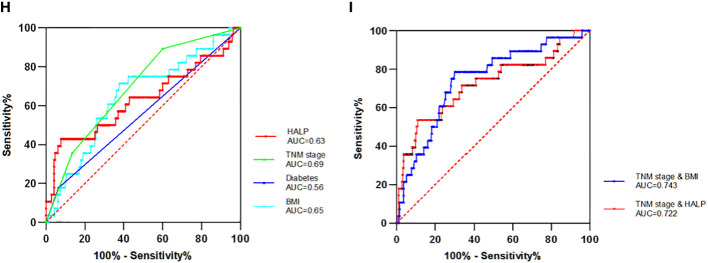
The Receiver operator characteristics (ROC) curve of single and combinate independent factors **(H)** Four single independent factors ROC curve. **(I)** The two combinations (TNM stage with BMI and TNM stage with HALP) with the largest AUC value. AUC denotes the area under the receiver operator characteristics curve.

The ROC curve analysis included the following four independent prognostic factors: HALP, diabetes, BMI, and TNM stage (**Figure 4**). The ROC curves demonstrated that HALP, diabetes, BMI, and TNM stage area under the curve (AUC) values were 0.63, 0.56, 0.65, and 0.69. Independent prognostic factors did not differ significantly except for stage and diabetes (P = 0.022).

To further search for indicators with higher predictive value, the four independent prognostic factors selected can be combined in pairs to calculate their AUC values. Finally, six sets of AUC values were obtained. The combinations were TNM stage and HALP, TNM stage and BMI, TNM stage and diabetes, HALP and diabetes, HALP and BMI, and BMI and diabetes. Among these, the combination of BMI and TNM stage had the maximum AUC value (AUC = 0.743), while that of HALP and TNM stage had the second largest value (AUC = 0.722). The AUC value of those combinations was higher than that of any single factor, which indicates that the combined factor has a better prognostic value for patients with EBC.

## Discussion

The various tumor stages are regulated by inflammatory indicators, nutritional status, and the immune response of the body (28). These factors can reflect the occurrence and development of cancer, indirectly reflect the body’s dependence on tumor drugs, and determine the body’s survival period in clinical practice. The preoperative NLR index has been reported to reduce the prediction of overall survival (OS) and progression-free survival (PFS) in patients with advanced tumors (5). The NLR >/= 2.2 before surgery has been reported to be significantly associated with retreatment after schwannoma resection (7). The high level of PLR was correlated with poor survival time (10), the results demonstrating a significant correlation between elevated PLR and poor prognosis in UC (11). An inflammation-based score, LMR index, is a significant predictor of better OS, DFS, and cancer-specific survival (13). Patients with LMR < 1.4 at the time of diagnosis had poorer PFS and OS than those with LMR > 1.4 in classical Hodgkin lymphoma (14). In clinical practice, a substantial proportion of cancer patients experience malnutrition as a result of the direct physiological effects of tumors, leading to issues such as indigestion, diarrhea, and the effects of anti-tumor treatments (29). the low level of preoperative PNI has worse OS and DFS in oral carcinoma. TNM stage and age had significant associations with low PNI, which indicates that the body’s nutritional status can also become an independent prognostic indicator for tumors (16). However, most clinical studies are limited to the impact of a single indicator on the occurrence, development, treatment, and prognosis of patients with tumors. As the research progresses further, using comprehensive indicators to predict the prognosis and treatment plans of patients with tumors is increasingly attracting the attention of researchers.

The HALP score cut-off value was different in the previous studies, with different studies selecting different determination methods. Combined with the previous research, almost all the studies used X-tile or ROC curves, with the cut-off value mostly between 15 to 50. According to a Mate analysis (30), 15/28 studies confirmed the value between 20 to 40. Currently, no evidence suggests that the HALP cut-off value was related to tumor type. A study (31) from the United States found that participants with a history of cancer had a lower HALP score than those without a malignancy, which also found that the median HALP scores varied dramatically by cancer type. Moreover, those with a history of ENT cancer had the lowest HALP scores (34.5), while those with a lung cancer history had the highest HALP scores (49.1). Another study (32) highlighted the heterogeneity of the “optimal threshold,” which meant the HALP score is the most predictive for individual cancer types in this threshold. It is possible to speculate that the ideal threshold for HALP is influenced by the extent of invasion in various types of malignant tumors. A single, specific value may not serve as the optimal threshold for all tumor types.

In this study, the prognostic significance of the HALP score was determined in EBC. The relationship between HALP score and postoperative DFS was similar to previous research results. K-M survival analysis demonstrated significant survival differences between high and low HALP groups (P<0.0001). HALP score<23.6 was associated with shorter RFS. Simultaneously, PLR>195.7 and LMR<4.9 were associated with shorter RFS. Different from previous studies, no evidence suggested that the level of NLR and PNI were related to survival time. According to multivariate Cox regression survival analyses, HALP score rather than LMR and PLR, diabetes, BMI, and TNM stage were independent prognostic indicators.

The overall diagnostic performance is assessed using the ROC curve. In this study, the ROC curve was not only used to predict single independent prognostic indicators but also the combinate factors. According to the AUC, the TNM stage had the highest value among single factors, followed by the BMI and HALP. The predictive value of prognosis is commonly evaluated using two or more indicators, while HALP combined with other prognostic indicators has not been applied to predict patients with EBC. There are six combinations of four indicators, where most combined indicators have higher AUC values than individual indicators with significant differences. The prognostic significance of the combinations of BMI with stage and HALP with stage was better than that of other combinations. This study also supports a narrative review (33), which reported that the advanced stage was linked to a higher risk of developing malnutrition that was an expression of the relationship between tumor burden, inflammatory status, reduced caloric intake, and malabsorption.

Inflammation, serving as a pivotal component of the innate immune response, represents a swift and precise reaction to foreign signals and tissue injuries. Nevertheless, as this response transitions from acute to chronic, the risk of cancer escalates. Several external factors, such as ultraviolet radiation, ionizing radiation, smoking, tobacco, alcohol, asbestos, and other carcinogens, are also contributors to the advancement of tumors. These factors are intrinsically linked to the invasion of various organs by inflammation. Taking measures to curtail the detrimental impact of inflammation on organs and tissues in the short term may potentially impede the onset and progression of tumors (28). Therefore, the inhibition of inflammation occurrence and dissemination can stand as a significant approach to chemotherapy, aiding in the prevention or postponement of tumor growth.

Our study had several limitations. First, this was a retrospective, single-center study, unable to avoid selection bias. Second, invasive breast cancer differs from high malignancy, invasion growth, and metastasis malignant tumors like NSCLC or PC, the 5-year or 10-year survival rate of EBC is relatively considerable after treatment. The median follow-up time in this study is approximately 54 months (4.5 years approximately), and insufficient follow-up time may lead to an overestimation of DFS or OS; therefore, this study can only provide a short-term prognostic reference value for patients with EBC. Extending follow-up time and monitoring the trend of changes dynamically may be needed to predict long-term value. Third, the four indicators are subject to many uncontrollable factors in clinical observation. Although diseases that may affect the patient’s blood biochemical indicators have been avoided, further improvement is desirable.

To conclude, HALP, as a novel biological indicator has not been completely studied, especially in predicting the survival value of patients with EBC. In this study, the HALP score was found to be significantly associated with worse RFS and was an independent prognostic factor for EBC. This score may predict the possibility of postoperative tumor recurrence or metastasis before surgery. The conjoint analysis of independent prognostic factors such as HALP, stage, diabetes, and BMI can obtain a higher predictive value than that of a single factor.

## Data availability statement

The raw data supporting the conclusions of this article will be made available by the authors, without undue reservation.

## Ethics statement

Ethical review and approval was not required for the study on human participants in accordance with the local legislation and institutional requirements. Written informed consent from the patients/participants or patients/participants’ legal guardian/next of kin was not required to participate in this study in accordance with the national legislation and the institutional requirements.

## Author contributions

ZZ: Conceptualization, Data curation, Formal analysis, Investigation, Methodology, Software, Writing – original draft. LX: Conceptualization, Funding acquisition, Resources, Supervision, Writing – review & editing.
