# Supplementary figures and images for: Prognostic significance of HALP score and combination of peripheral blood multiple indicators in patients with early breast cancer
Source: Front Oncol. 2023 Dec 12;13:1253895. doi: 10.3389/fonc.2023.1253895 (PMC10768851; doi:10.3389/fonc.2023.1253895)

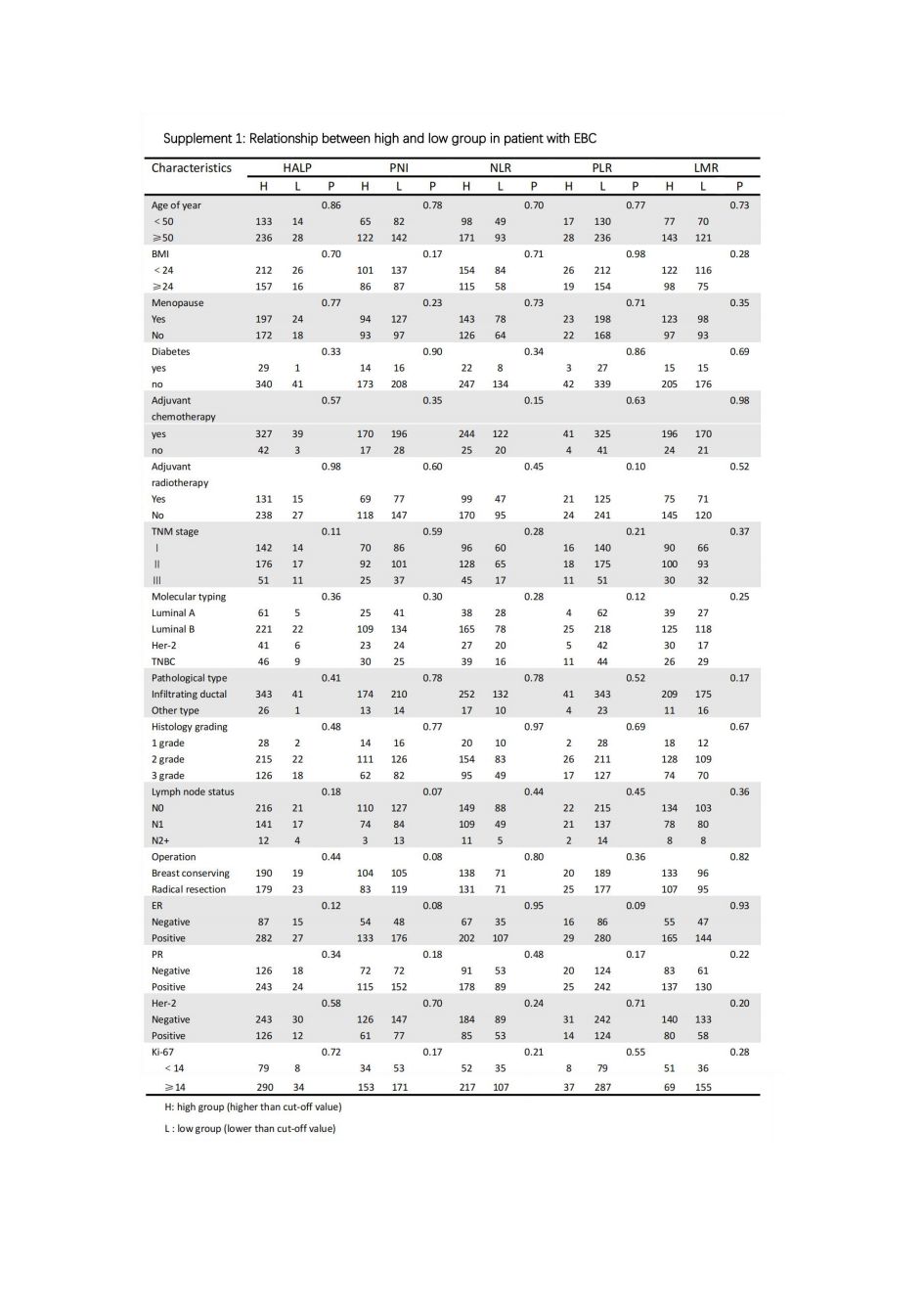

Supplement: Supplementary file 1 [file Image_1.jpeg]
